# Supplementary material for: Resolving the full spectrum of human genome variation using Linked-Reads
Source: Genome Res. 2019 Apr;29(4):635–45. doi: 10.1101/gr.234443.118 (PMC6442396; doi:10.1101/gr.234443.118)
Supplement: Supplemental Material [file supp_gr.234443.118_Supplemental_longranger-2.2.zip › longranger-2.2.tar.bz2!/lr2.2-public/lib/stan/cmdstan/stan/doc/api/html/classstan_1_1gm_1_1argument__parser.html]

Stan: stan::gm::argument\_parser Class Reference


|  |  |
| --- | --- |
|  | Stan  2.5.0  probability, sampling & optimization |


- Main Page
- Related Pages
- Namespaces
- Classes
- Files

- Class List
- Class Index
- Class Hierarchy
- Class Members

All Classes Namespaces Files Functions Variables Typedefs Enumerations Enumerator Friends Macros Pages

- stan
- gm
- argument\_parser

Public Member Functions |
Protected Attributes |
List of all members

stan::gm::argument\_parser Class Reference

`#include <argument_parser.hpp>`

|  |  |
| --- | --- |
| Public Member Functions | |
|  | argument\_parser (std::vector< argument \* > &valid\_args) |
|  | |
| int | parse\_args (int argc, const char \*argv[], std::ostream \*out=0, std::ostream \*err=0) |
|  | |
| void | print (std::ostream \*s, const std::string prefix="") |
|  | |
| void | print\_help (std::ostream \*s, bool recurse) |
|  | |
| void | print\_usage (std::ostream \*s, const char \*executable) |
|  | |
| argument \* | arg (std::string name) |
|  | |
| bool | help\_printed () |
|  | |

|  |  |
| --- | --- |
| Protected Attributes | |
| std::vector< argument \* > & | \_arguments |
|  | |
| bool | \_help\_flag |
|  | |
| bool | \_method\_flag |
|  | |

## Detailed Description

Definition at line 17 of file argument\_parser.hpp.

## Constructor & Destructor Documentation

|  |  |  |  |  |  |
| --- | --- | --- | --- | --- | --- |
| stan::gm::argument\_parser::argument\_parser | ( | std::vector< argument \* > & | *valid\_args* | ) |  |

inline

Definition at line 21 of file argument\_parser.hpp.

## Member Function Documentation

|  |  |  |  |  |  |
| --- | --- | --- | --- | --- | --- |
| argument\* stan::gm::argument\_parser::arg | ( | std::string | *name* | ) |  |

inline

Definition at line 207 of file argument\_parser.hpp.

|  |  |  |  |  |
| --- | --- | --- | --- | --- |
| bool stan::gm::argument\_parser::help\_printed | ( |  | ) |  |

inline

Definition at line 215 of file argument\_parser.hpp.

|  |  |  |  |
| --- | --- | --- | --- |
| int stan::gm::argument\_parser::parse\_args | ( | int | *argc*, |
|  |  | const char \* | *argv*[], |
|  |  | std::ostream \* | *out* = `0`, |
|  |  | std::ostream \* | *err* = `0` |
|  | ) |  |  |

inline

Definition at line 28 of file argument\_parser.hpp.

|  |  |  |  |
| --- | --- | --- | --- |
| void stan::gm::argument\_parser::print | ( | std::ostream \* | *s*, |
|  |  | const std::string | *prefix* = `""` |
|  | ) |  |  |

inline

Definition at line 138 of file argument\_parser.hpp.

|  |  |  |  |
| --- | --- | --- | --- |
| void stan::gm::argument\_parser::print\_help | ( | std::ostream \* | *s*, |
|  |  | bool | *recurse* |
|  | ) |  |  |

inline

Definition at line 148 of file argument\_parser.hpp.

|  |  |  |  |
| --- | --- | --- | --- |
| void stan::gm::argument\_parser::print\_usage | ( | std::ostream \* | *s*, |
|  |  | const char \* | *executable* |
|  | ) |  |  |

inline

Definition at line 157 of file argument\_parser.hpp.

## Member Data Documentation

std::vector<argument\*>& stan::gm::argument\_parser::\_arguments

protected

Definition at line 221 of file argument\_parser.hpp.

bool stan::gm::argument\_parser::\_help\_flag

protected

Definition at line 227 of file argument\_parser.hpp.

bool stan::gm::argument\_parser::\_method\_flag

protected

Definition at line 228 of file argument\_parser.hpp.

---

The documentation for this class was generated from the following file:

- src/stan/gm/arguments/argument\_parser.hpp


---

[ Stan Home Page ]

*© 2011–2014,
Stan Development Team.*
